# The Immunological Properties of Recombinant Multi-Cystatin-Like Domain Protein From *Trichinella Britovi* Produced in Yeast

**DOI:** 10.3389/fimmu.2019.02420

**Published:** 2019-10-11

**Authors:** Anna Stachyra, Anna Zawistowska-Deniziak, Katarzyna Basałaj, Sylwia Grzelak, Michał Gondek, Justyna Bień-Kalinowska

**Affiliations:** ^1^Witold Stefanski Institute of Parasitology, Polish Academy of Sciences, Warsaw, Poland; ^2^Department of Food Hygiene of Animal Origin, Faculty of Veterinary Medicine, University of Life Sciences in Lublin, Lublin, Poland

**Keywords:** *Trichinella britovi*, cystatin-like protein, *Pichia pastoris*, immunization, immunomodulation

## Abstract

Trichinellosis is a globally-distributed zoonotic parasitic disease caused by nematode worms of the genus *Trichinella*. One of the most common species of *Trichinella* known to affect human health is *T. britovi*; however, it is relatively poorly investigated. A thorough knowledge of the proteins expressed by *Trichinella* is important when developing immunological detection methods and vaccines and studying its interactions with the host. The present study uses the *Pichia pastoris* expression system to produce a soluble TbCLP antigen which induces strong antibody responses in the host during natural infection. Our results demonstrate the feasibility of TbCLP antigen production in yeasts, which are able to carry out post-translational modifications such as glycosylation and disulfide bond formation; they also indicate that the glycosylated TbCLP antigen had immunogenic effects in the tested mice and induced a mixed Th1/Th2 response, and was associated with a reduced larval burden after challenge with *T. britovi*. Subsequent *in vitro* stimulation of mice splenocytes revealed that TbCLP most likely possesses immunomodulatory properties and may play a significant role in the early phase of infection, affecting host immunological responses.

## Introduction

Trichinellosis is a common food-borne parasitic zoonosis worldwide. Infection occurs through the consumption of raw or inadequately-cooked meat containing *Trichinella* larvae: one of the most widespread intracellular parasitic nematodes affecting vertebrates (1, 2). The entire life cycle of the *Trichinella* parasite takes place in a single host after ingestion of infected muscle tissue. Parasitic infection can be divided into three separate antigenic stages: adult worms (Ad), newborn larvae (NBL), and muscle larvae (ML).

Within the *Trichinella* genus, the most widespread and most widely-investigated species has historically been *T. spiralis*. The parasite mainly occurs in domestic animals, and its circulation is traditionally associated with consumption of pig meat by humans. However, a range of other *Trichinella* species, such as *T. nativa, T. britovi, T. nelsoni, T. murelli*, and *T. pseudospiralis*, also circulate in sylvatic cycles and could also represent an accidental threat for human and domestic animals.

Of these species, *T. britovi* is the most widely distributed (1, 3). It can infect a wide range of mammalian hosts, mostly carnivores such as raccoon dogs, red foxes, and wolves, but is also known to invade various omnivores including wild boars, martens, badgers, and rodents (3–6). Trichinellosis is a widely-known public health hazard, especially in developing countries, but also represents an economic problem in the production of pork products and food safety. In contrast, developed countries tend to have a lower risk of trichinellosis associated with the consumption of pig-based products due to their high biosecurity standards and strict veterinary control in the pig farming and food processing industries. Currently, the most common source of trichinellosis in developed countries is through the consumption of game hunted for recreation: as it is not intended for sale, but only for private consumption, the meat is often not subject to veterinary inspection. Interestingly, while *T. spiralis* was found to predominate in samples of wild boar, a commonly consumed type of meat, *T. britovi* ML were also present in smaller numbers (7, 8). Hence, it is reasonable to assume that some cases of human trichinellosis may be caused by *T. britovi*, and numerous cases have already been confirmed (9–12). Therefore, to better understand the biology of the nematode and the host-parasite relationship, proteomical and immunological studies of *T. britovi* aimed at the identification of active proteins and potent antigens are needed.

Proteomical analysis (13, 14) and immunoscreening of cDNA expression libraries (15, 16) indicate that the multi-cystatin-like domain protein (named MCD or CLP) of *T. spiralis* is a highly antigenic protein. Its expression has been confirmed by RT-PCR in all developmental stages, with the highest level occurring in Ad. The protein was localized in the stichosome of Ad and ML and was also detected as a component of the excretory-secretory antigen (ES) of both Ad and ML (16–18). Hypothetical CLP was also recently detected by immunoblotting of somatic protein extract of *T. britovi* ML (19). Referred studies revealed, that this protein is produced in various stages of the parasite life cycle, after which it accumulates in the stichosome and is later released at specific stages of development as a component of the ES antigen, with its greatest release probably being at the intestinal stage (16).

Cystatins, inhibitors of cysteine proteinases, are a major class of parasitic nematode molecules with immunomodulatory properties that enhance production of the anti-inflammatory IL-10 cytokine and inhibit legumains, thus preventing MHC-II generation (20). Nematode proteinase inhibitors are generally known to play important functions at the host-parasite interface and are targeted by the immunological system. They control their own proteinase activity and that of the host, and weaken the immunological response by influencing antigen processing and presentation, cytokine production, and T-cell proliferation (21, 22).

Interestingly, the *Trichinella* CLP protein most likely has no cysteine protease inhibitory activity, and its function is unknown. The protein is classified within the cystatin superfamily, and demonstrates a similarity to family 3 kininogens: glycosylated and secreted proteins that are of relatively high mass and contain three family 2 cystatin-like domains (23). Robinson et al. (18) report that recombinant TsCLP produced in *E. coli* was not able to inhibit papain *in vitro*; the authors attribute this to the absence of two conserved motifs (QXVXG and PW in each cystatin domain), which are normally present in family 2 cystatins, and which form a structure that blocks the active site of the protease (24, 25). Instead of inhibitory activity, they propose that the protein can undergo self-processing and release single cystatin-like peptides to perform specific functions. Even so, the function of these peptides remains unknown. These low molecular weight isoforms of native CLP were observed in two-dimensional (2-D) analysis of ES antigen; the isoforms were also observed following expression of TsCLP in HeLa cells but not in *E. coli* (14, 16, 18), suggesting that CLP activity requires eukaryotic post-translational modification, such as the formation of disulfide bonds or glycosylation.

*Pichia pastoris* is a methylotrophic yeast used for expression of recombinant proteins; it is a particularly attractive host for this process due to its potential for methanol-dependent induction, triggered by the tightly-regulated AOX1 promoter (26). The system is useful for both large-scale and laboratory-level production of recombinant proteins, and can produce large amounts of the protein of interest. As a eukaryotic organism, *P. pastoris* ensures that the proteins undergo post-translational modification, and offers very economical handling and propagation. It is therefore an interesting alternative to other expression systems (27). Following eukaryote-specific post-translational modification, the produced recombinant proteins are generally highly similar to native proteins. Their disulfide bonds are correctly formed and preserved (28). The N-glycosylation profile is usually high mannose with no tendency to hyper-glycosylation and the oligosaccharides are often attached at the same positions as in natural glycoproteins (29, 30). A number of pharmacologically-active proteins are commercially produced in this expression system, e.g., hepatitis B vaccine antigen, human serum albumin, human IFNα, anti-RSV antibody and human insulin (source: https://pichia.com/). However, few studies have been published concerning the production of parasitic nematode proteins in this way (31–34), particularly *Trichinella* proteins.

CLP protein was identified as one of the reactive spots in our previous proteomic analysis of *T. britovi* antigens, based on a combination of 2-D immunoblotting of sera from infected pigs followed by LC-MS/MS analysis (19). It was therefore selected as a promising immunoreactive protein for further study, especially that the *T. britovi* homolog of CLP was yet not known. The aim of the present study was to achieve expression of the TbCLP antigen in a eukaryotic system, and to evaluate its potential diagnostic applications and protective efficacy against *T. britovi* infection in a mouse model.

## Materials and Methods

### *T. britovi* Parasite and Mouse Model

The reference strain of *T. britovi* (ISS002) had been maintained by several passages in male C3H mice at the Institute of Parasitology, PAS. Muscle larvae were used for challenge infection of C3H mice and as a source of genetic material for cloning. The larvae were recovered from the infected mice by HCl-pepsin digestion (35). The animals were housed in a temperature-controlled environment at 24°C with 12-h day-night cycles, and received food and water *ad libitum*.

### Sequence Analysis

The amino acid (aa) sequences were aligned using CLUSTALW. The N-glycosylation sites were predicted using NetNGlyc server (http://www.cbs.dtu.dk/services/NetNGlyc/).

### Cloning of Recombinant CLP Gene

*T. britovi* ML were obtained for total RNA isolation using Total RNA mini Plus kit (A&A Biotechnology). The RNA template was then used for cDNA synthesis with the Maxima First Strand cDNA Synthesis Kit for RT-qPCR (Thermo Scientific) according to the manufacturer's protocol. The cDNA coding the TbCLP gene without its signal peptide was amplified by PCR with CLP-specific primers (Forward: 5′-AGGCATCGATACAGATACTTGGTGAAAC-3′, Reverse: 5′-GCTCTAGAGCACATTCAACAGTTGACTTG-3′). Since the nucleotide sequence of *T. britovi* CLP was not known, primers were designed according to the nucleotide sequence of *T. spiralis* CLP (GenBank no. FR694976), whose amino acid sequence is thought to be very similar to the hypothetical sequence of TbCLP (GenBank no. KRY50178). The cDNA coding for TbCLP was sequenced and subcloned into the yeast expression vector pPICZαC with the His-tag sequence at the C-terminus (Invitrogen/Thermo). The correct reading frame of the recombinant plasmid was confirmed by DNA sequencing using vector flanking primers, 5′AOX1 and 3′AOX1.

### Expression and Purification of Recombinant CLP in *P. pastoris*

*Pichia pastoris* cells (X33 strain) were transformed with recombinant plasmids by electroporation. X33 transformant selection was performed using a medium containing Zeocin, and successful integration of the CLP gene into the *P. pastoris* genome was confirmed by PCR. The recombinant TbCLP with C-terminus His-tag was expressed by induction with 0.5% methanol for 24–72 h in 200 ml of Buffered Methanol-complex Medium (BMMY), and then purified by immobilized metal ion affinity chromatography using Protino Ni-NTA agarose (Macherey-Nagel). The protein samples were analyzed qualitatively by SDS PAGE and Western blotting, and their concentration was measured using Pierce BCA Protein Assay Kit (Thermo Scientific).

### Enzymatic Deglycosylation

Samples of recombinant protein purified from medium after 24, 48, and 72 h of induction were digested with Endo H (New England Biolabs) in denaturing conditions, according to the manufacturer's instructions. Deglycosylated rTbCLP were analyzed by SDS-PAGE.

### SDS-PAGE and Western Blot Analysis

SDS-PAGE was performed in 10% BisTris polyacrylamide gels. After electrophoresis, the gels were either stained with Coomassie brilliant blue or the proteins were transferred to a nitrocellulose membrane (Bio-Rad) for Western blotting. The recombinant protein was detected using monoclonal Anti-polyHistidine–Peroxidase antibody (diluted 1:4,000; Sigma). The proteins were viewed with Super Signal Western Pico Chemiluminescent Substrate (Thermo Scientific). Alternatively, rTbCLP was detected using sera from immunized or infected mice (1:200) and a secondary anti-mouse IgG antibody conjugated with HRP (1:8,000, Abcam).

### Determination of Recombinant Antigen Immunoreactivity by ELISA

A panel of *T. britovi* serum samples stored in our collection were used for testing rTbCLP as an antigen in ELISA; the samples had been obtained from pigs experimentally infected with 5000 *T. britovi* ML. Testing was performed on samples were taken at multiple time points post-infection (−4, 3, 6, 9, 13, 15, 17, 20, 24, 29, 36, 41, 45, 51, 55, 59, 62 days post-infection, dpi). ELISA plates were coated with 2 μg/ml of rTbCLP protein and incubated at 4°C overnight.

The pig serum samples were added at 1:100 dilution and the plates were incubated at 37°C for 1 h. Goat anti-pig IgG conjugated with peroxidase (Sigma) at 1:20,000 dilution was used for detection of CLP-specific antibodies. The enzymatic color reaction was generated using TMB substrate (3,3′,5,5′-Tetramethylbenzidine; Sigma), and the absorbance value was measured at 450 nm using a Synergy HT microplate reader (BioTek). The cut-off value of ELISA was evaluated on the basis of the average OD plus three standard deviations (SD) of *Trichinella-*free serum samples of pigs (36).

### Immunization and Challenge Infection

Eight-week old male C3H mice were divided into three groups of 12 animals each. The vaccine group was immunized subcutaneously with 25 μg of rTbCLP emulsified with the adjuvant Alhydrogel (InvivoGen) in a total volume of 100 μl (antigen/Alhydrogel = 75/25 v/v). The mice were boosted with the same dose after 1 week. The control groups were injected with PBS or PBS+Alhydrogel using the same regimen. 1 week after the final vaccination, six mice from each group were sacrificed, and their blood and spleens were harvested for immunological tests. The remaining six mice from each group were challenged orally with 500 *T. britovi* ML. 7 weeks (48 days) after infection, all mice were sacrificed, the blood and spleens were harvested, and the *T. britovi* muscle larvae were recovered by HCl-pepsin digestion. Protective immunity was calculated according to the number of ML recovered from the vaccinated group compared with those from the PBS group. Sera from all mouse blood samples were isolated and frozen at −20°C for further analysis.

### Determination of Serum-Specific Antibodies

TbCLP-specific antibodies (IgG as well as subclasses IgG1 and IgG2a) in serum samples of vaccinated mice were measured by indirect ELISA, with rTbCLP as coating antigen, at 1 week after final immunization and 7 weeks after challenge infection. ELISA plates were coated with 2 μg/ml of rTbCLP protein and incubated at 4°C overnight. Following this, mouse serum samples at 1:200 dilution were added and incubated at 37°C for 1 h. Then, the plates were incubated with HRP-conjugated antibody goat anti-mouse IgG, IgG1, or IgG2a (1:80,000 or 1:60,000, Abcam) for detection of CLP-specific antibodies. The enzymatic color reaction was generated and the cut-off value of ELISA was evaluated as described in the present study.

### Cytokine Analysis

To measure the specific cellular response, the spleens were harvested from vaccinated animals 1 week after final immunization and 7 weeks after challenge infection.

The spleens were pooled into pairs taken from two randomly-selected mice within the same group; following this, the splenocytes were disassociated using a 70 μm cell strainer and then suspended in complete RMPI medium (Biowest). In order to lyse the erythrocytes, the splenocytes were incubated in 5 ml RBC lysis buffer (Thermo Scientific) for 10 min. The cell suspension was centrifuged at 250 × g at room temperature for 7 min. The cell pellets were washed in RPMI medium and then resuspended in complete RPMI medium containing 10% FBS and penicillin/streptomycin (Biowest); following this, the cells were then counted.

For the cytokine stimulation assay, the splenocytes were seeded in a 24-well culture plate (Corning) at 5 × 10^6^ cells per well in 1,000 μl medium. The cells were stimulated with 15 μg/ml rTbCLP and incubated for 72 h at 37°C in a humidified atmosphere of 5% CO_2_. Cells stimulated with 5 ug/mL Concavalin A were included as positive controls. Non-stimulated cells were included as negative controls. After 72 h, the cells were pelleted by centrifugation at 1,000 × g for 10 min and the supernatants were collected to measure cytokine production. Samples containing the supernatant were tested for levels of IL-2, IL-4, IL-10, IFN-γ using a Mouse Th1/Th2 uncoated ELISA kit (Invitrogen/Thermo Scientific).

### Statistical Analysis

Statistical analysis was performed using Statistica 6 software (StatSoft). Data were expressed as means ± standard deviation (SD). Differences among groups were analyzed by one-way analysis of variance (ANOVA). A value of *p* < 0.05 was considered significant.

## Results

### Cloning, Expression, and Characterization of Recombinant TbCLP Protein

**Figure 1:**
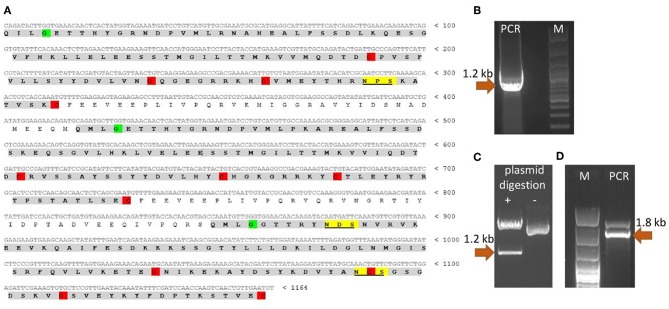
Cloning of TbCLP coding sequence. **(A)** Cloned cDNA sequence and predicted amino acid sequence of TbCLP. Three cystatin-like domains are highlighted in gray [according to Robinson et al. (18)]. Conserved cysteines, that form disulfide bonds are highlighted in red. Conserved glycines are highlighted in green. Predicted N-glycosylation sites are underlined and highlighted in yellow. **(B)** PCR product after amplification of TbCLP cDNA. **(C)** Restriction digestion of recombinant plasmid containing target insert. **(D)** PCR product after screening of *P. pastoris* transformants. Correct DNA fragments are marked with arrows and the molecular mass is indicated.

Since the nucleotide sequence of *T. britovi* CLP was not known, PCR primers were designed according to nucleotide sequence of *T. spiralis* CLP. TsCLP (GenBank no. CBX25716) shares 91.5% identity with the hypothetical sequence of TbCLP (Genbank no. KRY50178) derived from conceptual translation of genomic data (37). As the amino acids in regions including the primers sequences was found to be identical in TbCLP and TsCLP, the DNA sequence for TbCLP without its signal peptide was successfully amplified and subcloned into the expression vector (Figure 1). Sequencing revealed full compatibility between the hypothetical and cloned TbCLP, with regard to the amino acid sequence, and the nucleotide sequence was 96.2% identical with that of the TsCLP gene. The sequence of the cloned TbCLP without the signal peptide is given in Figure 1A.

The theoretical molecular weight of the His-tagged TbCLP was predicted as 47.3 kDa, and the molecular weight of a single cystatin-like domain was 12 kDa. NetNGlyc server analysis of the amino acid sequence identified three potential N-glycosylation sites in the protein chain: one in the first cystatin-like domain and another two in the third cystatin-like domain.

CLP gene expression was induced in the X33 *P. pastoris* strain with methanol. In preliminary experiments, 0.5% methanol was added every 24 h until the final time of induction was reached. Further analyses indicated that a single induction with methanol and 24-h cultivation yielded the best rTbCLP level. The recombinant His-tagged protein was purified using affinity chromatography under native conditions.

**Figure 2:**
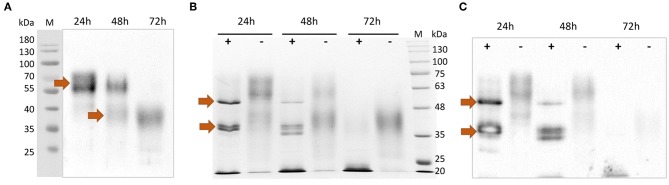
Analysis of purified rTbCLP. **(A)** Anti-His Western blot of protein purified from medium after 24, 48 and 72 h of induction. High molecular weight glycoisoforms (55–70 kDa) and low molecular weight glycoisoforms (35–45 kDa) are marked with arrows. **(B)** SDS PAGE and **(C)** Anti-His Western blot after deglycosylation of rTbCLP with Endo H. Protein samples were incubated with Endo H enzyme (+) or without Endo H (–) in denaturing conditions. Deglycosylated full form rTbCLP (48 kDa) and the shorter isoforms (35–38 kDa) are marked with arrows.

After purification, the rTbCLP protein appeared to be present as various different glycoisoforms, manifesting as blurred, smear bands with molecular weights of ~55–70 and 35–45 kDa on Western blot (Figure 2A). Interestingly, different protein forms were observed after 24, 48, and 72 h of induction: high molecular weight glycoisoforms were visible after 24 and 48 h of induction, and only low molecular weight glycoisoforms were visible after 72 h of induction. In order to examine the N-glycosylation of recombinant CLP protein, an Endo H digestion procedure was used. Deglycosylation with Endo H reduced the molecular mass of rTbCLP (Figures 2B,C) and confirmed that the observed variation in protein masses was caused by changes in glycan content. After the release of N-linked carbohydrates from the protein, the upper band corresponded well with the predicted mass of full length TbCLP (48 kDa), and the lower bands corresponded with the shorter isoforms (35–38 kDa), which may be created by post-translational cleavage events. After 24 and 48 h of induction, the full form of the protein was visible, as well as several shorter forms (Figures 2B,C). After 72 h of induction, the full form of protein was no longer visible, and the total amount of protein seemed very low, indicating that the whole pool of rTbCLP expressed in *P. pastoris* had been processed. The protein was taken after 24 h of induction, purified and used in subsequent experiments.

### rTbCLP Recognition by Sera From *T. britovi* Infected Pigs

**Figure 3:**
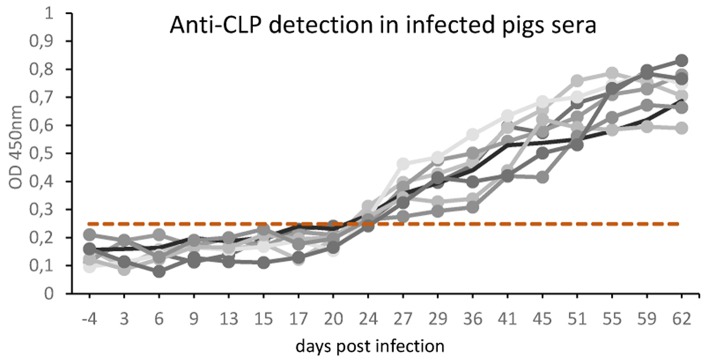
Anti-*Trichinella* IgG levels in pigs infected with *T. britovi* by ELISA with rTbCLP. Pigs (*n* = 8) were experimentally infected with 5,000 ML of *T. britovi* and sera were collected prior to infection (−4 dpi) and 3, 6, 9, 13, 15, 17, 20, 24, 29, 36, 41, 45, 51, 55, 59, 62 dpi. The cut-off value was evaluated on the basis of the average OD plus three SD of *Trichinella*-free serum samples and is marked as a dashed line. All individuals were found positive after 24 days post-infection.

The results in Figure 3 represent the antibody kinetics in eight pigs that were experimentally infected with 5000 ML *T. britovi* using rTbCLP. The ELISA results demonstrate that seroconversion was detected at 24 days post-infection. The moment of detection was earlier than when using a commercial PrioCHECK® *Trichinella* Antibody ELISA Kit (Thermo Scientific) containing ES *Trichinella* antigen (data not shown). The results confirm the antigenic properties of rTbCLP protein and indicate that rTbCLP is a potential antigen to be used in future experiments for detection of *Trichinella* infection in animals.

### Humoral Antibody Response Induced by Immunization of Mice With rTbCLP Formulated With Alhydrogel

**Figure 4:**
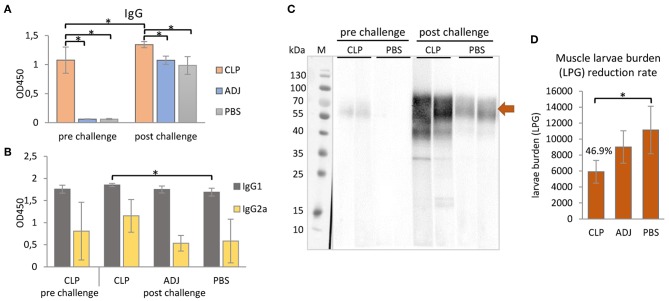
Mouse immune responses to the immunization of rTbCLP. **(A)** Anti-TbCLP total IgG level and **(B)** anti-TbCLP subclasses IgG1 and IgG2a level in sera of immunized mice, measured by ELISA after final immunization and challenge infection, respectively. **(C)** Anti-TbCLP total IgG in sera of experimental mice, detected by Western blot. Two randomly selected sera from each group were used for immunodetection of rTbCLP (3 μg/lane). Specific signal for 55–70 kDa form is marked with arrow. **(D)** Muscle larvae burden reduction rate (%) in three experimental mice groups after challenge infection. Experimental groups: CLP—group injected with rTbCLP+adjuvant, ADJ—group injected with adjuvant, PBS—group injected with PBS. Significant differences are marked with asterisks. Bars represent mean values from six individuals (*n* = 6) ± SD.

Purified rTbCLP was tested for immunogenicity in a mouse model. The total IgG level in serum was first evaluated in immunized and control groups; following this, the humoral antibody responses in the immunized mice were assayed by ELISA, with rTbCLP as a coating antigen (Figure 4A).

The results indicate that anti-TbCLP IgG antibodies were elicited by the immunization of mice with rTbCLP+adjuvant. Specific anti-TbCLP IgG were detected in all serum samples from the immunized group; however no anti-TbCLP antibody response was detected in the mice injected with adjuvant or PBS alone. Although anti-TbCLP antibodies were present in sera of all experimental groups after the challenge infection, a significantly higher level of specific anti-TbCLP was observed in the group of mice immunized with rTbCLP+adjuvant than the two control groups.

A relatively high level of IgG1 and a moderate level of IgG2a were observed following induction by rTbCLP. After the challenge infection with *T. britovi*, all groups demonstrated a high level of IgG1; however, the mice injected with rTbCLP+adjuvant displayed a significantly higher level than the PBS control group. In contrast, moderate levels of anti-TbCLP subclass IgG2a were detected in all tested groups, but no significant differences were detected between groups due to the high level of individual variation (Figure 4B). The presence of anti-TbCLP antibodies was also confirmed by Western blot, using sera from the rTbCLP+adjuvant and the PBS control groups after immunization and after challenge infection. Induced IgG were able to give specific signal as showed at Figure 4C.

### Cytokine Profiles of Stimulated Splenocytes From Mice Immunized With rTbCLP

**Figure 5:**
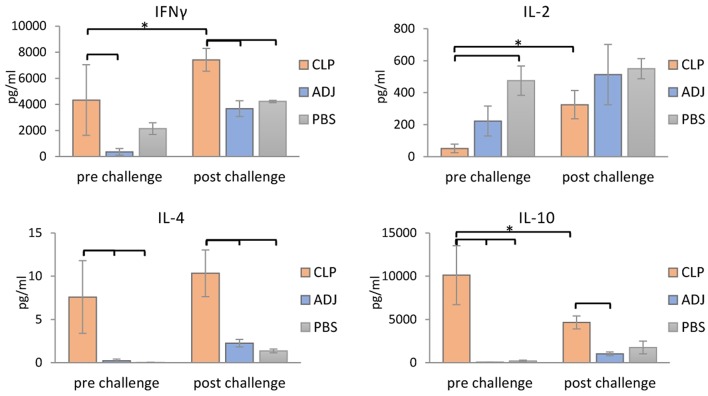
Cytokines detection in supernatants of stimulated splenocytes of the immunized mice. Supernatants harvested after 72 h of incubation were analyzed for detection of secreted cytokines IFNγ, IL-2, IL-4, and IL-10 using Mouse Th1/Th2 uncoated ELISA kit. Experimental groups are indicated as previously described. Immunized groups significantly different from corresponding control groups are marked with empty brackets, while corresponding immunized groups significantly different from each other are marked with asterisks. Bars represent means ± SD from three splenocytes cultures (*n* = 3) each prepared from two pooled spleens from mice from the same group.

To further confirm that a Th1/Th2 mixed response was induced following rTbCLP vaccination, the levels of selected cytokines (IFNγ, IL-2, IL-4, and IL-10) were measured in the supernatants of stimulated splenocyte cultures. In the case of Th1, higher levels of IFNγ were identified in splenocytes from mice immunized with rTbCLP+adjuvant than controls treated with adjuvant or PBS alone, with significantly lower levels observed in the adjuvant controls. However, after challenge infection with *T. britovi*, significantly higher levels of IFNγ were found in the rTbCLP+adjuvant group than the others (Figure 5). In contrast, IL-2 levels were significantly lower in the rTbCLP+adjuvant cultures than the PBS control group. After infection, these levels was still lower in the cultures from immunized mice than controls; however, these differences were not statistically significant.

Clearer differences were visible in case of the Th2 cytokines: the levels of IL-4 and IL-10 were significantly higher in the supernatants of the immunized mice than in the two control groups, in which the cytokine levels were at the limit of detection (Figure 5). After infection, the level of IL-4 in the immunized group was at a similar value than before infection, but the level of IL-10 was clearly lower than before infection. These results confirm that immunization with TbCLP triggered a mixed Th1/Th2 response.

### Protective Immunity Induced by rTbCLP

The protective response induced by rTbCLP against *T. britovi* infection was investigated in experimental C3H mice. Seven weeks (48 days) after infection, all mice (six per group) were sacrificed and *T. britovi* muscle larvae were recovered from individual mice. The results revealed a significantly different reduction rate (46.9%) between the immunized group and the PBS control group (Figure 4D).

## Discussion

Multi cystatin-like domain protein (CLP) is promising immunoreactive protein used in studies to control trichinellosis. Although *T. spiralis* CLP has previously been cloned and used for immunization (16, 18, 38), the properties of *T. britovi* CLP are generally unknown, and only genomic data were available of its hypothetical sequence. Our findings indicate a great degree of similarity between the CLP of *T. britovi* and that of *T. spiralis*. Fortunately, the target gene could be successfully amplified by PCR (Figure 1B) as the annealing regions in the coding sequences of *T. britovi* and *T. spiralis* were found to share a high degree of similarity. The two nematodes are closely related, and TsCLP and TbCLP share 91.5% identity in their amino acid sequences and 96.2% identity for their nucleotide sequences.

To evaluate the potential of *P. pastoris* as an expression host for production of a recombinant *T. britovi* CLP, the pPICZαC/CLP vector was constructed (Figures 1C,D) and expressed in X33 strain cells. The expressed rTbCLP displayed fuzzy, smear bands on Western blot (Figure 2A); it was difficult to precisely determine the mass of the detected protein bands and their mass appeared to vary depending on the duration of induction, i.e., 24, 48, or 72 h. It is known that in some cases protein expression in *P. pastoris* demonstrates heterogenic glycosylation, resulting in its protein population displaying diverse structural heterogeneity (30). Within a cell, two molecules of the same protein may present different oligosaccharide profiles, even when they have been exposed to the same glycosylation machinery. Such variation is typically derived from differences in the type, length and identity of the oligosaccharide at a given glycosylation site. The glycoprotein may also vary with regard to site occupancy.

In case of rTbCLP, great variation can be seen in protein subpopulations. Similar results were previously described by Teh et al. (39), where human erythropoietin (EPO) expressed in the *P. pastoris* system also demonstrated broad smear band in Western blot. EPO is a native glycoprotein (40), as is TbCLP and family 3 cystatins. Fractionation of rEPO followed by PNGase F and Endo H treatment found that the observed variations in molecular mass, manifested as the broad smear observed on the membrane, were caused by variations in glycan content: the deglycosylated protein demonstrated a sharp band with the predicted molecular mass. It is important to emphasize that the recombinant EPO was fully functionally active, despite the observed variation in glycosylation. In our study, deglycosylation with Endo H also resulted in the rTbCLP displaying sharp bands, and revealed the presence of high molecular weight and low molecular weight isoforms (Figures 2B,C).

A previous study has found recombinant TsCLP to possess different isoforms (18). Two such isoforms were found to be expressed in Hela cells, one of 49 kDa and another of 38 kDa; these were described respectively, as the full-length protein and the shorter isoform. Subsequent incubation at different pH values revealed that a further two low molecular weight isoforms (35 and 38 kDa) were visible in the pH range 4–8, and these bands were most prominent at pH 5. The authors suggest that the CLP undergoes pH-dependent auto-processing, resulting in the release of individual cystatin-like peptides (18).

This conclusion supports our observation that the amount of full length TbCLP decreases during induction in *P. pastoris*, with only low molecular-weight isoforms being observed after 72 h. It is known that in *Pichia* fermentation, the pH decreases while feeding from a carbon source, as the catabolic activity leads to acid production, potentially resulting in greater processing of the secreted rTbCLP into individual peptides. For this reason, only the protein isolated after 24 h of induction was used in the following experiments. In the future, to scale-up protein expression, it will be necessary to optimize the induction conditions, particularly the control of medium pH, to prevent early processing. Nonetheless, it is important to emphasize that in contrast to the rCLP previously produced in bacteria (16, 18), the *P. pastoris* system yielded an active, functional form of rTbCLP: native CLP is most probably secreted as a glycosylated protein containing disulfide bonds, and the initial full length form is further transformed into the shorter forms, these being the single cystatin-like peptides with specific biological functions, due to post-translational modification.

Our previous 2-D immunoblotting and LS-MS/MS analyses revealed some spots that reacted with the sera of *T. britovi* infected pigs; these were identified as a hypothetical protein highly similar to a multi-cystatin-like domain protein precursor from *Trichinella spiralis*. The molecular mass of the spots ranged from 45 to 50 kDa, which corresponded with the expected mass of CLP (19). To confirm this immunological reactivity, ELISA tests were performed to determine the level of anti-rTbCLP IgG in infected pig sera and seroconversion was detected at 24 dpi (Figure 3). However, high background values were observed and further optimization of test procedures is needed before the rTbCLP antigen can be used in diagnostics of anti-*Trichinnella* antibodies in serum samples.

Infection with *Trichinella* is initially characterized by the induction of a Th1 response at the beginning of the intestinal phase; however, the extensive dissemination of newborn larvae results in a shift to a Th2 response. The Th2 state is protective and results in parasite expulsion. *Trichinella* worms rapidly develop to maturity and reproduce before the Th2-mediated expulsion eliminates all adult forms from the gut. Thus, during the intestinal phase, the immune response is mixed, i.e., Th1/Th2, with an initial predominance of the Th1 response shifting to that of the Th2 response (41). In the present study an analysis of mouse humoral immune responses revealed high total IgG levels in serum; in addition, while IgG1 levels were clearly higher than those of IgG2a after vaccination with rTbCLP, IgG2a was also elicited, suggesting that the mixed Th1/Th2 immune response was triggered by immunization with rTbCLP (Figures 4A,B). The mixed immune response was further verified by the levels of Th1 (IFN-γ, IL-2) and Th2 cytokines (IL-4, IL-10) observed in the spleen cell cultures of immunized mice following stimulation by rTbCLP protein. Although all tested groups of mice displayed unique cytokine profiles, all cytokines in the immunized cultures were significantly elevated compared to controls, while IL-2 was significantly suppressed (Figure 5).

IFNγ is a signature cytokine of the adaptive immune response, and the main cytokine associated with Th1. High levels of IFNγ were detected in samples taken from groups after immunization and after challenge infection; however, the level of IFNγ was almost twice as high in the challenged group. This finding suggests that rTbCLP effectively induced the Th1/cellular response, which was then boosted after infection.

IL-2 plays a dual role in immunological system: it is a potent T cell growth factor associated with protective immune responses, but also influences immune tolerance and the downregulation of inflammation. The function of IL-2 *in vivo* is complex, and its influence on the immune response is not only dependent on its presence or absence, but also the level of IL-2 receptor (IL-2R) signaling that takes place (42, 43). The effect of immunization and splenocyte stimulation on IL-2 production *in vitro* is hard to interpret, but it may be related with CLP possessing certain immunomodulatory properties, which are currently unknown; these could affect IL-2R signaling pathways and the cytokine network in immune cells, resulting in inhibited IL-2 secretion and/or possibly augmented IL-2 consumption. Interestingly, our results correspond with those of an immune response analysis of *Trichinella* infection described by Yu et al. (44), where IL-2 level was found to be significantly downregulated in mouse sera during the intestinal and larvae migration phases compared to uninfected controls. Other tested cytokines were elevated or unchanged compared to controls during the respective infection phases, and only IL-2 exhibited downregulation during the early phase of infection. Suppression of IL-2 production may also be partially explained by the release of high amounts of IL-10, since IL-10 can inhibit production of IL-2 (45). Interestingly no other cytokine secretion was inhibited in our experiment, indicating that this phenomenon may be somehow related with the dichotomous role played by IL-2 and IL-2R in self-tolerance and immunity.

IL-4 is closely associated with the humoral (Th2) and anti-helminthic responses (46); as it regulates B-cell immunoglobulin secretion, it was not surprising that it was observed at high levels in cultures following immunization compared to naïve controls. In a similar way to IFNγ, the highest level of IL-4 was observed in the immunized and infected group, demonstrating that vaccination very effectively primed Th2 responses against the *T. britovi* parasite; this response was boosted further after infection, and the findings are in agreement with our IgG level analysis. However, the fact that the amounts of IL-4 found in all experimental supernatants were relatively small might be explained by the fact that IL-4 has a short half-life, and the cells were cultured for 72 h (47).

IL-10 is a well-known anti-inflammatory cytokine, and that during parasite infection, it plays a major role in parasite immunomodulation by suppressing immune responses. More interestingly, the main factor inducing the production of IL-10 by APCs, particularly macrophages, during infection are thought to be nematode cystatins (20, 48). Although the exact mechanism by which these cystatins activate IL-10 production in host cells is unknown, it is most probably independent of protease inhibitory activity, and is likely related to their structural features and receptors engagement. TbCLP was previously assumed to lack protease inhibitory activity; therefore, due to its conserved structure, it may modulate the host immune system by inducing the production of anti-inflammatory IL-10. Indeed, our findings indicate the presence of very high amounts of IL-10 in the supernatants of stimulated splenocytes. The level of IL-10 was the highest in the immunized group; however, these levels were significantly lower in the immunized and infected group, suggesting that impact of recombinant protein alone was very strong, but this influence was balanced by the broad immune response to infection. Interestingly, production of IL-4 and IL-10 by splenocytes was much weaker after *T. britovi* infection than after rTbCLP immunization. This could be possibly explained by the fact that the infection was in its late phase: the splenocytes were harvested 48 dpi, when the parasite is at the convalescent phase of the life cycle.

rTbCLP vaccination resulted in a 46.9% larvae reduction (Figure 4D); this was a similar benefit to one reported in a previous study of recombinant CLP in *T. spiralis* (16), where mice vaccinated with rTsCLP exhibited a significant reduction in muscle larvae burden. However, the previous experiment was conducted using Freund's Complete Adjuvant (CFA) and the antigen was administered intraperitoneally. These are very harsh immunization conditions, and CFA is known to cause some concerns in animal usage, due to its toxicity and painful reaction; even so, it is often used in mice (49, 50). In contrast, our present study used an alum adjuvant, which is non-toxic and approved for use in humans (51). It obtained good results, suggesting that yeast-derived antigen is superior to bacteria-derived antigens, as the milder immunization conditions yielded a similar immunoprotective effect. It is important to mention that, as far as we are aware, all recombinant *Trichinella* antigens tested so far have been produced in a bacterial expression system and may not have been fully active (52–56). To better characterize the functions of the proteins of interest, a eukaryotic expression system, such as the yeast expression chosen in this study, should be used.

## Conclusions

Immunization with the active full form of rTbCLP resulted in high immunogenicity in tested mice and a significant reduction of larvae burden after experimental infection. The recombinant protein was produced in *Pichia* cells; this approach ensures that the protein is further processed into individual cystatin-like peptides via post-translational modification. Furthermore, it can be assumed that cytokine profiles of the presented splenocytes are both resultants of the immune response against recombinant TbCLP antigen administered as an experimental vaccine, as well as the product of the immunomodulation of immune cells by this antigen, resulting from its biological function. The molecular events triggered by rTbCLP as an antigen, or as a modulator, could be opposing or synergistic. This dichotomy is manifested in the observed imbalance of IL-2 and IL-10, two cytokines involved in immunosuppression (57). However, it should be remembered that our findings illustrate just a small part of the host response to immunization and infection. Future research on the immunomodulatory properties of TbCLP may allow for a better understanding of its function, and further studies on the usage of TbCLP for the purposes of diagnosis and vaccine development would be also desirable.

## Data Availability Statement

All datasets generated for this study are included in the manuscript/supplementary files.

## Ethics Statement

The procedures were approved by the First Local Ethical Committee for Scientific Experiments on Animals in Warsaw, Poland (resolution no.: 020/2016, 23 March 2016) and the Second Local Ethical Committee for Scientific Experiments on Animals in Lublin, Poland (resolution no.: 77/2015, 7 July 2015). All efforts were made to minimize suffering. All applicable international, national and/or institutional guidelines for the care and use of animals were followed. In order to collect material from infected and/or immunized mice, the animals were humanely euthanized by cervical dislocation.

## Author Contributions

AS and JB-K conceived and designed the experiments and analyzed the data. AS, AZ-D, and KB were involved in cloning. AS performed protein expression and analysis and immunological study. AS, J-BK, and SG participated in mice immunization and *in vitro* experiments. MG supplied the pig sera. AS wrote the manuscript. JB-K, AZ-D, and KB participated in reviewing and editing the manuscript. JB-K supervised the manuscript. All of the authors have read and approved the final manuscript.

### Conflict of Interest

The authors declare that the research was conducted in the absence of any commercial or financial relationships that could be construed as a potential conflict of interest.
